# Malaria from hyperendemicity to elimination in Hekou County on China–Vietnam border: an ecological study

**DOI:** 10.1186/s12936-017-1709-z

**Published:** 2017-02-07

**Authors:** Jian-Wei Xu, Jian-Jie Li, Hong-Ping Guo, Shu-Wei Pu, Shu-Mei Li, Rong-Hua Wang, Hui Liu, Wei-Jia Wang

**Affiliations:** 1 Yunnan Institute of Parasitic Diseases, Yunnan Provincial Centre of Malaria Research, Yunnan Provincial Key Laboratory of Vector-borne Diseases Control and Research, Puer, 665000 China; 2 Honghe Prefecture Centre for Disease Prevention and Control, Mengzi, China; 3 Hekou County Centre for Disease Prevention and Control, Hekou, China

## Abstract

**Background:**

Malaria control and elimination are challenged by diversity and complexity of the determinants on the international border in the Great Mekong Sub-region. Hekou, a Chinese county on the China–Vietnam border, was used to document Chinese experiences and lessons for malaria control and elimination.

**Methods:**

The design was an ecological study. Malaria burden before 1951 and procedures of 64 years (1952–2015) from malaria hyperendemicity to elimination are described. Single and bilinear regression analysis was utilized to analyse the relationship between the annual malaria incidence (AMI) and gross domestic product (GDP), urbanization rate, and banana planting area (BPA).

**Results:**

There was a huge malaria burden before 1951. AMI was reduced from 358.62 per 1000 person-years in 1953 to 5.69 per 1000 person-years in 1960. A system of primary health services, comprising three levels of county township hospitals and village health stations maintained malaria control and surveillance activities in changing political and social-economic settings. However, potential under-reported of malaria and market-oriented healthcare led to a malaria epidemic in 1987. Strong political commitment reoriented malaria from a control to an elimination programme. High coverage of malaria intervention and population access to intervention was crucial for malaria control and elimination; meanwhile, AMI was closely associated with socio-economic development, correlation coefficients (R) −0.6845 (95% CI −0.7978, −0.6845) for national GDP, −0.7014 (−0.8093, −0.7014) for national urbanization rate and −0.5563 (−0.7147, −0.3437) for BPA.

**Conclusions:**

Multifactor, including political commitment, effective interventions, social and economic development and changing ecological environment, and the complicated interactions between these factors contribute to malaria elimination in Hekou County.

**Electronic supplementary material:**

The online version of this article (doi:10.1186/s12936-017-1709-z) contains supplementary material, which is available to authorized users.

## Background

Malaria is a parasitic disease that is transmitted by female *Anopheles* mosquitoes [1]. About 214 million (range 149–303 million) new cases of malaria occurred worldwide in 2015 [2]. Now, an ambitious new goal has been set by the World Health Organization (WHO) to reduce the global malaria burden by 90% by 2030 [1], and to eliminate malaria by 2030, as well as to eliminate falciparum malaria by 2025 in all countries of Greater Mekong Sub-region (GMS) [3]. Malaria is closely correlated between any two countries such as China and Myanmar along their common border [4, 5]. Control and elimination of malaria are challenged by diversity and complexity of the determinants in the GMS [6, 7], especially on the international border [6, 7]. Malaria movement across international borders is one of the major obstacles to malaria elimination [8–10].

Hekou County was a malaria hyperendemic areas on the China–Vietnam border. Malaria had ever been the most life-health-threaten diseases historically in this region. Socio-economic and environmental characteristics have changed greatly, and malaria is from hyperendemicity to elimination from 1952 to 2015 [11]. Now it is the first county to achieve malaria elimination its international border in southern China, and its achievement was certified by Yunnan Province on 4 November, 2015. Although nowadays the Chinese National Malaria Elimination Action is stable and moving forward [12, 13], related experiences and lessons are not well documented and reported. This paper describes the malaria burden before 1951 and the 64-year period (1952–2015) of procedures, from malaria hyperendemicity to elimination, analyses the impact of political commitment, interventions, social economic development, and ecological environment, on malaria and then discusses experiences and lessons learnt from global malaria eradication.

## Methods

### Study site

**Fig. 1:**
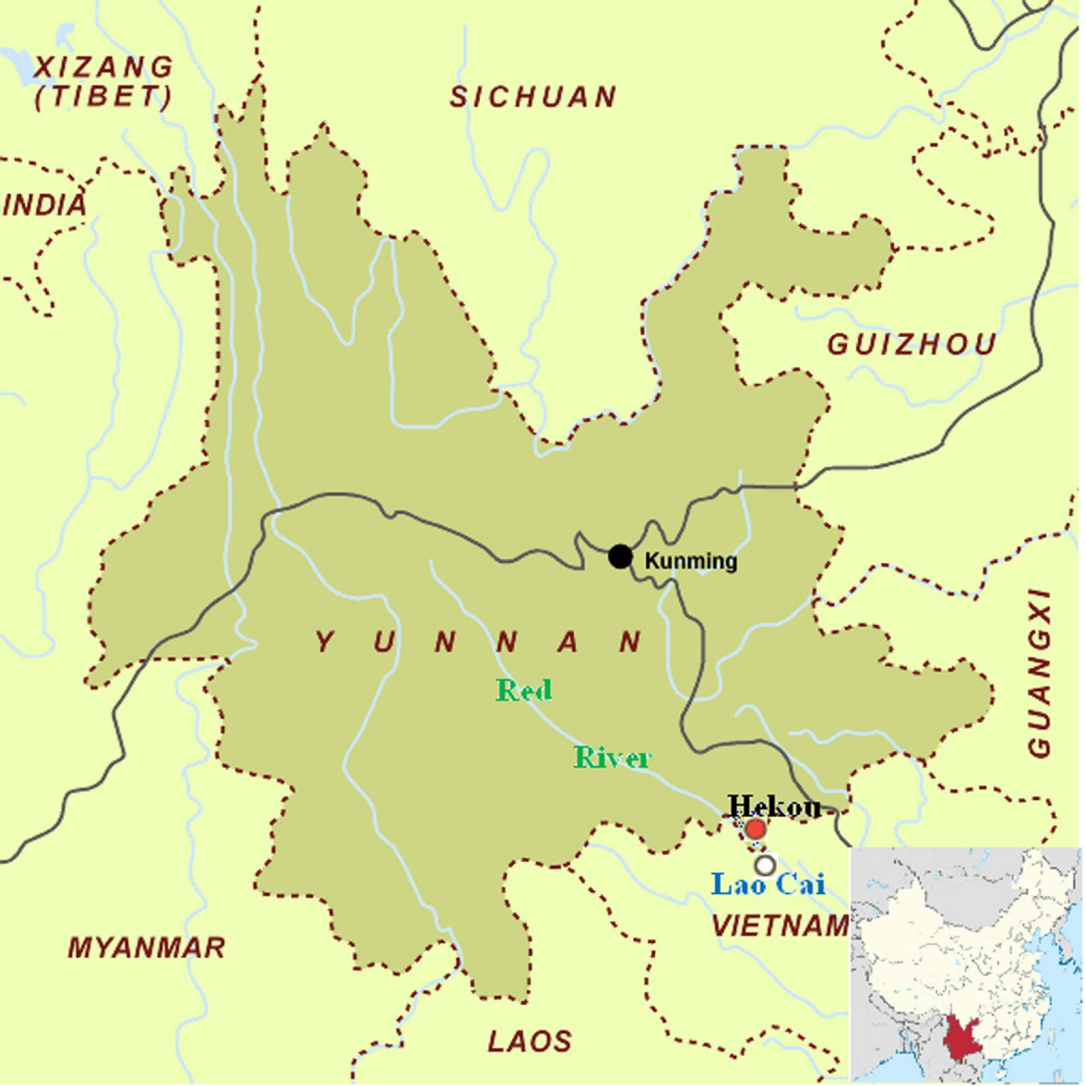
Map of the study site and neighbouring region

The study was carried out in Hekou County (103°23′–104°17′E, 22°30′–23°02′S; altitude from 76.4 to 2354.1 m), which is located at Red River Valley, southeast of Yunnan Province, China, and shares a border of 193 km with Vietnam (Fig. 1). The county has a population of 104,609 people (2010) living in an area of 1332 km^2^, with a tropical monsoon rainforest climate and an annual mean temperature of 23.0 °C [14]. *Anopheles minimus* is the primary malaria vector [15, 16]. Indigenous malaria transmission was interrupted at Hekou County in 2011, and it passed the provincial elimination assessment in 4 November, 2015.

### Study design

The study design was an ecological study. Malaria burden before 1951 and the 64-year dynamics (1952–2015) from malaria hyperendemicity to elimination are described. Association between malaria incidence and socio-economic development and environmental change are analysed by single and bilinear regression.

### Data resource and collection

All available paper records related to malaria surveillance and control activities were reviewed at Hekou County Centre for Disease Control and Prevention (CDC) for collecting data of malaria cases and intervention activities from 1952 to 2007. Chinese Information System for Disease Control and Prevention (CISDCP) began to cover all counties of China from 2008 [17], thus, data on malaria cases and intervention from 2008–2015 were obtained from CISDCP. National and provincial gross domestic product (GDP) and urbanization rate came from the website of the National Bureau of Statistics of the People’s Republic of China. Banana planting area (BPA) was from paper records of Hekou County Bureau of Statistics. Qualitative data was collected by semi-structured, in-depth interviews (SDI) with senior malaria control staff who had worked at Hekou County CDC, Honghe Prefecture CDC and Yunnan Institute of Parasitic Diseases (YIPD) [18, 19]. In addition, almost all available literature on political, social and economic development, the malaria situation and control related to Hekou County were also reviewed.

### Data analysis

**Fig. 2:**
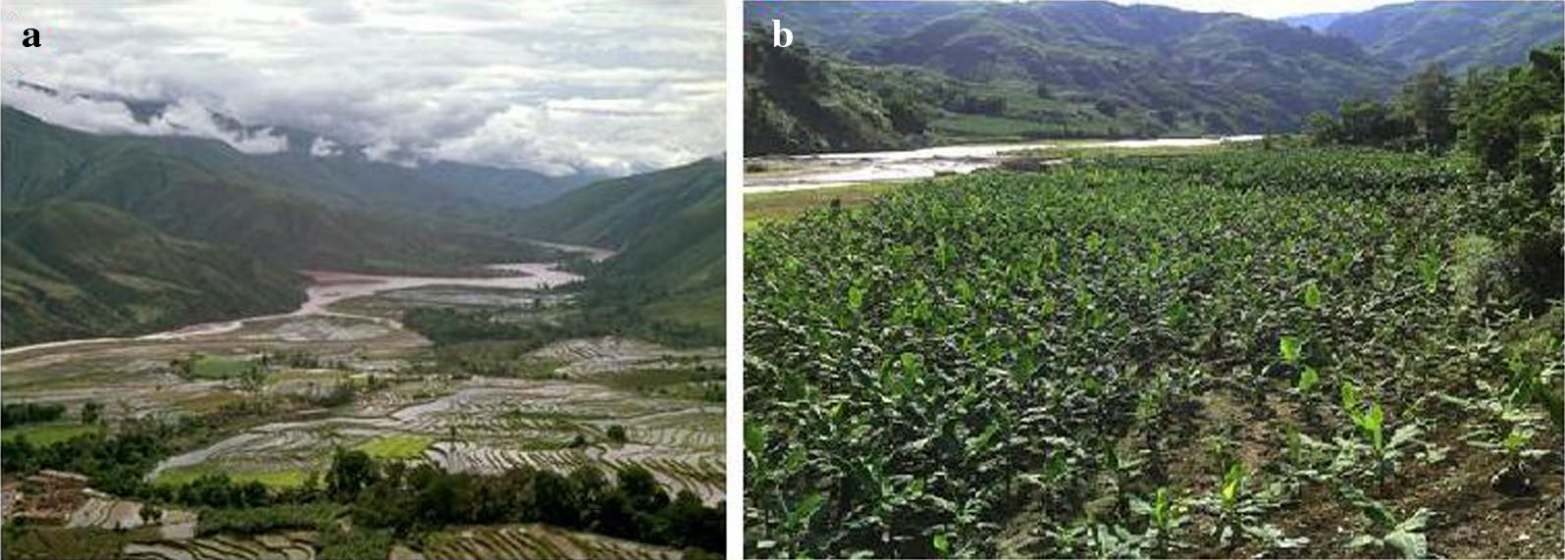
Changed breeding environment for mosquito larvae due to growing bananas

Before 2010, species of malaria parasites were not registered clearly. A combination of clinical symptoms and results of presumptive treatment with anti-malarial drugs were some of the definitions for malaria cases at that time. In the study, annual malaria incidence (AMI) was used to replace annual parasite incidence as the outcome variable, so the AMI included both microscopy confirmed cases and cured cases by presumptive treatment. The amount and impact of intervention activities were described for each stage in the process from hyperendemicity to elimination, although the quantitative relationship between AMI and intervention activities was not done because of unavailability of sequential data. In linear regression analysis, independent variables for social and economic development were national GDP in billions of China Yuan (CNY) and urbanization rate (%). In Hekou County, banana growing is major part of the economy, which has changed the environment in terms of malaria transmission. Transition from paddy field to banana plantation reduces breeding sites for mosquito larvae, and insecticides used in growing bananas kills mosquitoes. In this reason, BPA (hectares) was used as an independent variable for environmental changes (Fig. 2). In order to obtain a more normal distribution of data, all outcome and independent variables were transformed into common logarithms, and then a linear regression analysis was used to analyse relationships of AMI respectively with GDP, urbanization rate, and BPA from 1952 to 2013 [20]. The number of malaria cases during 2014–2015 was zero, so data of the 2 years could not be taken into the analysis model. The rapid development of society and economy has improved health information system and reduced the data bias from 2000 to 2015, so the analysis model was conducted for years 2000–2013 again. In order to learn the impact of socio-economic development and environmental change, a bilinear regression analysis was conducted to on AMI association with national GDP and BPA, and with urbanization rate and BPA for years 2000–2013 [20].

## Results

### Malaria burden and interventions, 1901–1951

A formal health service system for malaria surveillance and control in China was not available before 1951. Despite significant differences from different data sources, e.g., the number of deaths from malaria as 12,000 in the report from the French Indo-China Railway Construction Company, to 60,000–70,000 in Chinese literature during the period of the Yunnan railway construction, these records indicated a large number of deaths and frequent outbreaks in Hekou County (Table 1). In 1939, Yunnan Commission of Malaria Control and six Provincial Institutes for Malaria Control (PIMC) were established in the Province, and PIMC 6 was allocated in Hekou County. PIMC 6 organized outreach teams visiting communities to treat malaria, promote use of bed nets, and treat mosquito larvae breeding sites with emerald green (copper acetoarsenit). However, these interventions were terminated because of Japanese military invasion to Yunnan in 1942.

**Table 1:** Historical record of malaria burden and interventions in Hekou County, Hehong (Red River) Prefecture, Yunnan Province, China from 1901 to 2015

Year	Reporters	Description of malaria burden and interventions	Data resources
1901 to 1907	Sheng Zuyan	French Indo-China Railway Construction Co started railway construction in Yunnan in 2001. Sheng, an officer from Chinese Qing Dynasty Government reported in 1907 “Based on inspection to construction of the railway line, at least 60,000–70,000 workers died of malaria, hungry and violence”	The report to Emperor Guangxu of the Qing Dynasty
1910	French Indo-China Railway Construction Co	During construction of Yunnan railway, 12,000 deaths, 80 of those fatalities French	Manual of Yunnan Railway Line
1910	Archives of Yunnan Province	From 2004 to 2010, about 300,000 workers were recruited from Yunnan, Guangdong and Sichuan Province to build Yunnan–Vietnam Railway, 60,000–70,000 deaths, most due to malaria	Beginning and ending of Yunnan–Vietnam Railway
1917	Hekou Anti-epidemic Station	A puppet show team had 24 actors and actress, 23 fatalities due to malaria	Archives of Hekou County
1940	Hekou Anti-epidemic Station. Yang et al.	A malaria outbreak in Hekou Town with a population of 3000: about 20 people died each day, total 851 deaths in 1940. There were 120 soldiers of Kuomintang Army and 13 soldiers survived. In 1940, a cross-sectional survey conducted by Dr. Zheng Zuyou documented 18.18% parasite prevalence and 47.30% enlarged spleen rate	Archives of Hekou County. Yunnan Malaria
1944	Hekou Anti-epidemic Station	In 1944, a team of Kuomintang Army settled in Hekou County, one half of them died associated with malaria	Archives of Hekou County
1949	Hekou Anti-epidemic Station	496 of 614 inhabitants from 123 families died in four villages (Yaoshan Feihui, Mali, Shibanzhai, Dazhai), none survived in 19 households in a malaria outbreak	Archives of Hekou County
1952	Hekou Anti-epidemic Station	Establishment of malaria control system, initiation of control interventions	Archives of Hekou County
1953	3rd Military Malaria Control Team of Yunnan Province	6591 malaria cases reported, AMI 358.62 per 1000 person-years, 89.32% of these cases *were P. falciparum*	Archives of Hekou County
1963	Hekou County Anti-epidemic Station	Hekou Anti-epidemic Station was established for malaria control. 288 malaria cases reported, AMI reduced to 8.02 per 1000 person-years	Archives of Hekou County
1969	Hekou County Anti-epidemic Station	County Anti-epidemic Station combined into County Hospital. No malaria case could be reported because of Great Cultural Revolution Campaign	Archives of Hekou County
1987	Hekou County Anti-epidemic Station	A malaria epidemic in June 1987, 2331 malaria cases, AMI 34.41 per 1000 person-years and 5 deaths. Outpatients of Xinjie Township Hospital treated 1531 malaria cases and malaria accounted for 32.81% hospitalization patients from June to December. A cross-sectional study documented 43.17% (180/417) of slide positivity rate, 87.78% (158/180) of *P. vivax*, 11.67% (21/180) of *P. falciparum*, 0.56% (1/180) of mixed infection among febrile patients, and 10.03% (258/2570) parasite prevalence among residents. Entomological investigation collected 539 anopheline mosquitoes, including 244 (45.3%) *An. minimus* (local primary vector), man-biting rate was 81.3 mosquitoes per person-night	Archives of Hekou CDC
1988	Hekou County Anti-epidemic Station	Intensive efforts were undertaken to control the outbreaks in 1987. MDA to 10,227 inhabitants for radical cures from January to March (dry and low transmission season). Chemoprophylaxis delivered to 22,091 local residents and 2085 migrants from June to November (wet and high transmission season). 726,868 m^2^ of indoor wall surfaces sprayed with DDT and 6262 bed nets treated with deltamethrin. Malaria cases reduced to 1018 and no fatality. AMI reduced to 14.78 per 1000 person-years	Archives of Hekou CDC
1995 to 2002	Hekou County Anti-epidemic Station	As a result of the epidemic, Hehong Prefecture top AMI among 17 prefectures in Yunnan Province. Government gave special attention to malaria and invested funding for anti-malarial drugs and insecticides. By 2002, number of malaria patients reduced to 75 cases and AMI to 0.98 per 1000 person-years	Archives of Hekou CDC
Jan 2003 to Jun 2010	Hekou County CDC	From 2003 to 2008, under support of the first round of GFATM for malaria, 19,607 febrile cases diagnosed by microscopy, 427 malaria cases confirmed and treated with anti-malarial drugs, 4963 courses of presumptive treatments delivered to suspected malaria patients, 1313 courses of radical cure treatments (RCT) to people with malaria attack history last year, 4414 courses of RCT to hyperendemic communities, 13,619 courses of preventive treatments to people at high risk, IRS conducted for 52,635 households in 1316 villages and 8466 bed nets impregnated with insecticide From 2007 to June 2010, under support of the fifth round of GFATM for malaria, 23,688 febrile cases were diagnosed by microscopy, 91 malaria cases confirmed and treated, 7278 courses of presumptive treatments given to suspected malaria patients, 1523 courses of RCT to people with malaria attack history last year, 4529 courses of RCT to hyperendemic communities, 4003 courses of preventive treatments to people at high risk, IRS conducted for 14,784 households in 464 villages and 10,886 bed nets impregnated with insecticide. 16 malaria cases were detected by 2008	Archives of Hekou County CDC
Jul 2010 to Dec 2015	Hekou County CDC	Efforts were taken in surveillance, focus investigation and rapid response since malaria elimination action launched on 1 July, 2010. 10 microscopy stations were strengthened, 13,943 and 2309 febrile cases were test by microscopy and RDT respectively, the foci of 4 confirmed malaria (2 *P. vivax* and 1 *P. falciparum*) and 780 suspected malaria cases investigated, and active detections conducted in 85 originally hyperendemic villages. Health education and social mobilization promote treatment-seeking and use of preventive measures. Mechanism of collaboration and information exchange established between Hekou County of China and Lao Cai Province of Vietnam. 300 suspected Vietnamese malaria cases tested by microscopy and one *P. vivax* cases detected treated. Meanwhile, a cross-sectional study tested 400 Vietnamese inhabitants for malaria. Vector mosquitoes were investigated in 2 Vietnamese villages. The last local infection was vivax malaria detected from a 76 years old man on 22 June, 2011. From then on, there was no indigenous case. Yunnan Province certified Hekou County malaria free in November, 2015	Archives of Hekou County CDC

### Interventions and incidence, 1952–2009

**Fig. 3:**
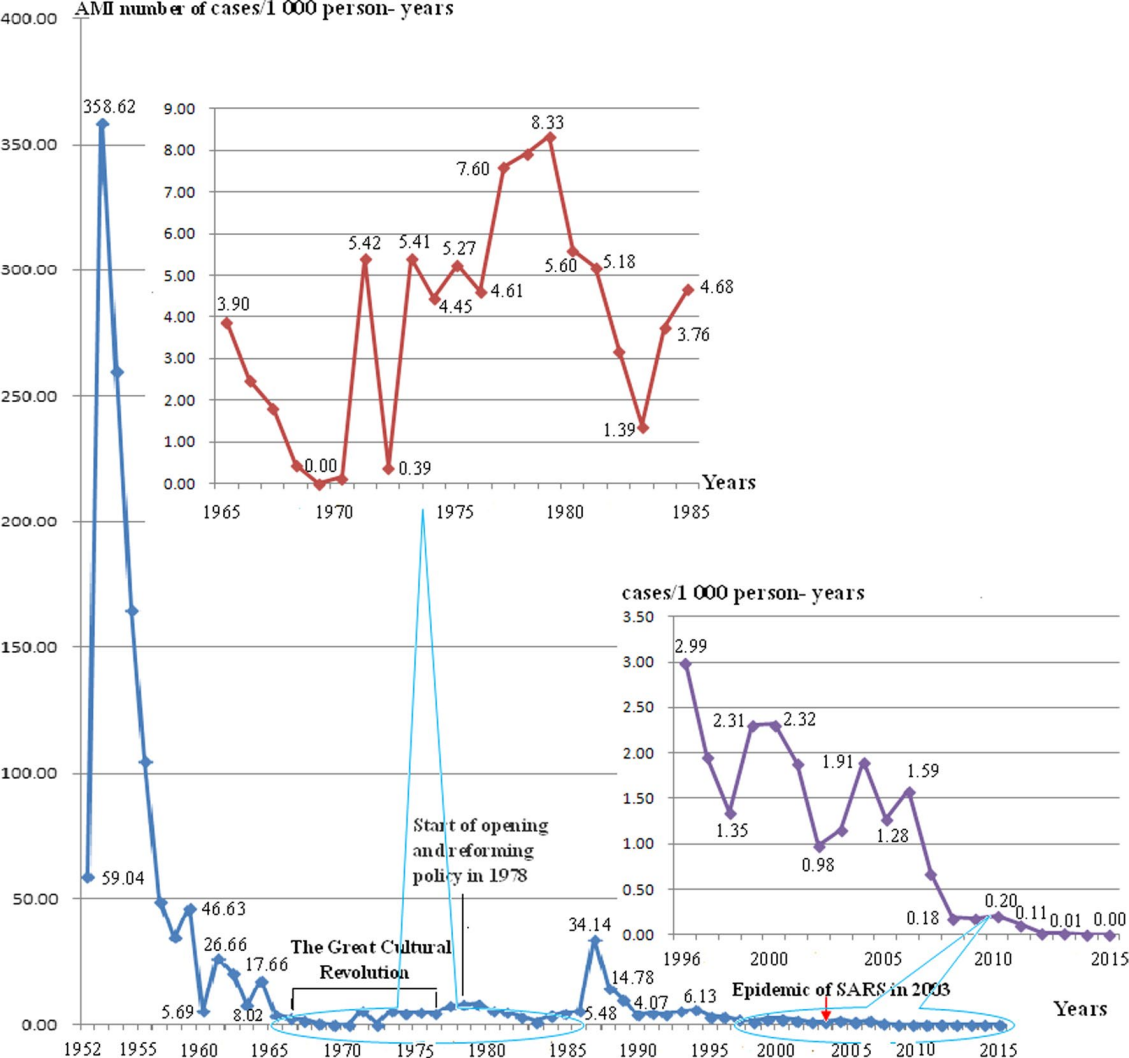
Annual malaria incidence (AMI) in Hekou County from 1952 to 2015

*1952–1962* Malaria investigation and control activities were initiated in 1952. According to results of SDI from former senior malaria control staff, a malaria control station (MCS) was established to recruit and train health staff in early 1952, and then a malaria control structure comprised of technical officers from military medicine departments and health sector of central and local governments, community health staff (CHWs) and village malaria control workers (VMCWs) was created. The leaders were political officers who managed technical officers, and technical officers trained, guided and managed CHWs who trained, guided and managed VMCWs. One VMCW was in charge of five to ten households to administer drugs and spray insecticides. Malaria investigation and control activities were initiated in the winter of 1952. Two treatment methods were utilized: (1) presumptive treatment was given to people with enlarged spleens and malaria attack episodes in prior 6 months; (2) intermittent preventive treatment (IPT), once a week, was administered to others. Methods for vector control were treatment of larval breeding sites with insecticides in the low-transmission dry season from December to April, and indoor residual spraying (IRS) in the high-transmission rainy season from May to November; in addition, community mobilization was conducted for environmental management. During those years of low supplies, drugs and insecticides were used as long as they were available. Occasionally obtainable drugs were quinacrine hydrochloride, quinine sulfate, paludrine and/or plasmoquine. Obtainable insecticides were dichlorodiphenyltrichloroethane (DDT) or hexachlorocyolohexane (C6H6C6). These interventions reduced AMI from 358.62 per 1000 person-years (89.32% *Plasmodium falciparum* of cases) in 1953 to 5.69 per 1000 person-years in 1960 (Table 1; Fig. 3).

*1963–1977* In Hekou County in October 1963 the MCS became the Anti-epidemic Station (AES). This was a formal system of primary health care services comprising of three levels of county hospital and AES, township hospitals and village health stations for communicable diseases. The system conducted vector control and parasite treatment regularly, and radical cure treatment in low-transmission seasons. Chloroquine and pamaquine were introduced for malaria treatment in this period. However, anti-malarial efforts were halted by the Great Cultural Revolution from 1966 to 1976. The AES was combined into Hekou County Hospital in 1968, and then detached from the hospital in 1971. This led to under-reporting of malaria; no malaria was reported in 1969 (Table 1; Fig. 3).

*1978–2002* A policy of reform and stimulation of economic activities and population movement challenged the public health system. Internal and transnational population migration increased transmission and the spread of malaria. Less attention was paid by governments, and health staff, including CHWs, left for better economic income, which led to continued under-reporting of malaria. When a malaria epidemic broke out in 1987: AMI increased to 34.41 per 1000 person-years and five malaria-related deaths in the year; Honghe Prefecture had the highest number of malaria cases among 17 prefectures in Yunnan Province. Ultimately, deterioration of the malaria situation brought a political will to malaria control. Hehong Prefecture Government invested enough funding for anti-malarial drugs and insecticides. Additionally, artemisinin was registered in China in 1986. Introduction of artemisinin saved the life in control of the epidemic, and then oral and injective artemisinin monotherapy was recommended as the first line drugs for treatment of *P. falciprum* malaria and artemisinin-based combination therapy (ACTs) as the second line treatment in China. By 2002, AMI was lowered to fewer than one per 1000 person-years (Table 1; Fig. 3).

*2003–2009* The Chinese government gave a stronger political commitment to public health after the epidemic of severe acute respiratory syndrome (SARS) in 2003. The Hekou County AES turned into Hekou County CDC. Malaria vector control, surveillance and treatment interventions were strengthened across the county. In addition, the Global Fund to Fight AIDS, Tuberculosis and Malaria (GFATM) grants strengthened core interventions. Intensive effort was made to seek and treat malaria to reduce under-reporting. In 2008, a cross-sectional survey screened 3900 residents by microscopy from 15 originally hyperendemic villages, in which no parasite carrier was detected. By 2008, only 16 malaria cases were reported, and the AMI was lowered to 0.18 per 1000 person-years (Table 1; Fig. 2).

### Elimination, 2010–2015

China re-oriented its malaria programme from control to elimination in July 2010. In order to look for infections and to interrupt transmission, the focus was shifted from high levels of coverage of interventions to an emphasis on completeness and timeliness of activities. Methodology of reactive case detection (RACD) was used in eliminating malaria. The ‘1-3-7’ approach was introduced for guiding and monitoring case reports, investigation and response, respectively: reporting of malaria cases within 1 day (24 h), their confirmation and investigation within 3 days, and the appropriate public health response to prevent further transmission within 7 days. Since 2012 no indigenous malaria infections have been detected in Hekou. Only one imported malaria case was found in 2012 and 2013 respectively, and no any malaria case in 2014 and 2015. Yunnan Provincial Health and Family Planning Commission (HFPC) received an application for evaluating malaria elimination from Honghe Prefecture HFPC in September, 2015. A team comprising experts of health system and information management, surveillance, monitoring and evaluation, laboratory diagnosis quality assurance, epidemiology and entomology, conducted an evaluation of malaria elimination based on “China National Guidelines for Malaria Elimination Evaluation and Certification”. Yunnan Province certified the county malaria-free in 4 November, 2015 (Table 1; Fig. 3).

### Relationship between AMI and socio economic development and environmental change

**Fig. 4:**
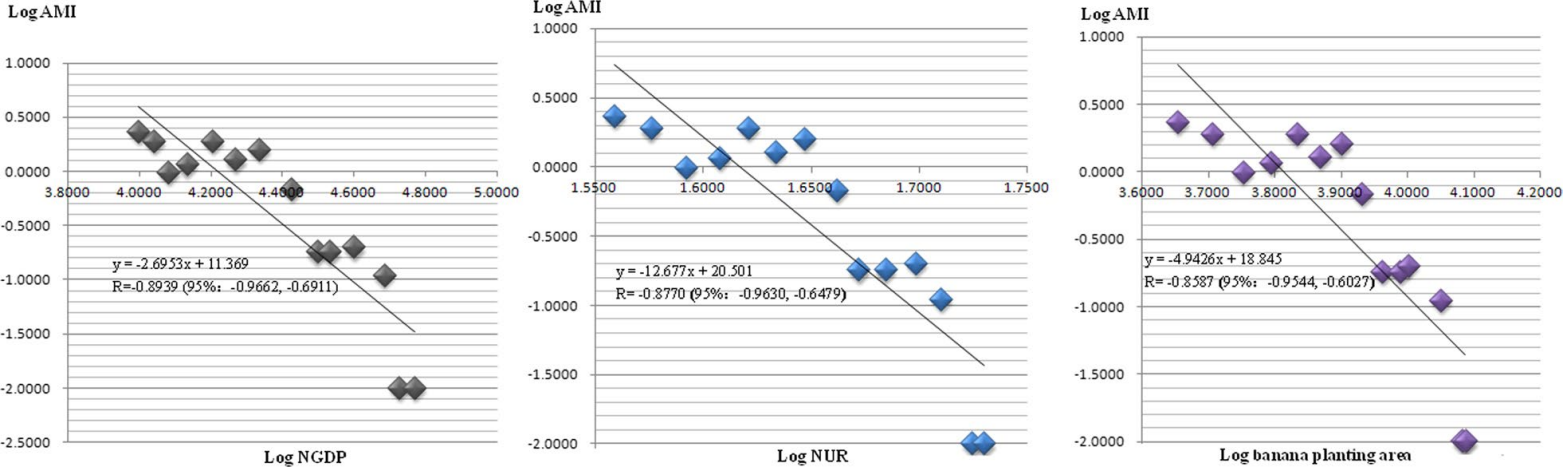
Annual malaria incidence (AMI) and national gross domestic product (NGDP) versus AMI and national urbanization rate (NUR) versus AMI and banana planting area in Hekou County during 2000–2013

Results of linear regression analysis indicated that AMI was negatively associated with national GDP (NGDP), national urbanization rate (NUR) and BPA. Correlation coefficients (R) were −0.6845 (95% CI −0.7978, −0.6845) for NGDP, −0.7014 (−0.8093, −0.7014) for NUR and −0.5563 (−0.7147, −0.3437) for BPA (Table 2). Analogous results were obtained for Yunnan Provincial GDP (YNGDP) and Yunnan Provincial urbanization rate (YNUR) (Table 2). Years 2000–2015 was a period of rapid socio-economic development and environmental change in China, and the data malaria cases was less biased than prior to 2000–2015. The relationship between AMI and these independent variables was more closely associated in this period (Fig. 4). When a bilinear regression analysis was conducted, R was −0.9103 (−0.9716, −0.7345) for NGDP and BPA, and −0.9055 (−0.9700, −0.7216) for NUR and BPA (Table 2). All these results show that the AMI had a negatively strong correlation with socio-economic development and environmental change.

**Table 2:** Results of linear regression analysis between annual malaria incidence (AMI) and independent variables in Hekou, Yunnan Province, China

Relationship between	R (95% CI)	R^2^ (95% CI)	P value
1952–2013			
AMI and NGDP	−0.6845 (−0.7978, −0.6845)	0.4685 (0.4685, 0.6366)	<0.0001
AMI and NUR	−0.7014 (−0.8093, −0.7014)	0.4919 (0.4919, 0.6550)	<0.0001
AMI and YNGDP	−0.6802 (−0.7949, −0.6802)	0.4627 (0.4627, 0.6319)	<0.0001
AMI and YNUR	−0.6892 (−0.8011, −0.6892)	0.4750 (0.4750, 0.6417)	<0.0001
1958–2013			
AMI and BPA	−0.5563 (−0.7147, −0.3437)	0.3095 (0.1181, 0.5108)	<0.0001
2000–2013			
AMI and NGDP	−0.8939 (−0.9662, −0.6911)	0.7990 (0.4776, 0.9336)	<0.0001
AMI and NUR	−0.8770 (−0.9630, −0.6479)	0.7692 (0.4198, 0.9274)	<0.0001
AMI and YNGDP	−0.8933 (−0.9660, −0.6895)	0.7980 (0.4755, 0.9332)	<0.0001
AMI and YNUR	−0.8919 (−0.9656, −0.6859)	0.7954 (0.4705, 0.9323)	<0.0001
AMI and BPA	−0.8587 (−0.9544, −0.6027)	0.7374 (0.3633, 0.9109)	<0.0001
AMI and NGDP with BPA	−0.9103 (−0.9716, −0.7345)	0.8286 (0.5394, 0.9440)	<0.0001
AMI and NUR with BPA	−0.9055 (−0.9700, −0.7216)	0.8199 (0.5207, 0.9410)	<0.0001

*R* correlation coefficient, *R* ^*2*^ coefficient of determination, *95% CI* 95% confidence interval, *NGDP* national gross domestic product, *NUR* national urbanization rate, *YNGDP* Yunnan Provincial gross domestic product, *YNUR* Yunnan Provincial urbanization rate, *BPA* banana planting area

## Discussion

The purpose of the paper is to describe the malaria burden before 1951, the procedures from hyperendemicity to elimination of malaria for 64 years (1952–2015) and related socio-economic and environmental factors in a county on the China–Vietnam border, and discuss Hekou’s experiences and lesson implication for global malaria eradication.

Stable political and social status is important for effective malaria interventions. Paper-based records and interviewees’ descriptions showed that Hekou was part of the hyperendemic areas before the establishment of People’s Republic of China. Malaria burden during Yunnan–Vietnam railway construction is one of the earliest highlighted records on malaria burden in China. In spite of the fatality difference in Chinese and French records, the two records document a heavy malaria burden (Table 1). In those war-ridden years, it was difficult to carry out effective malaria intervention. The new government of China put people’s wellbeing in an important position and gave political commitment to malaria control. In the context of shortage of armamentarium, medicine and human resources, the effective collaboration between military and civil medicine sectors set up an effective system for malaria prevention and treatment in a short time, which reduced malaria burden significantly (Fig. 3). Importantly, the system of malaria control expanded into a primary health care system (PHCS). Similarly, malaria elimination in Sri Lanka documented the importance of political will for malaria control. Strong governmental commitment eliminated malaria in this underdeveloped country [21].

The PHCS played important role in the history of malaria control. The PHCS in rural areas was called rural cooperative medical system (RCMS) that was developed under collective financing and public support from the People’s Commune. Health personnel and facilities were organized on a three-tier system (county, commune and village). ‘Barefoot doctors’ were the backbone of the RCMS and their emphasis was on prevention. They were trained in both therapeutics and prophylaxis, both western and traditional Chinese medicine. In addition, women barefoot doctors learn midwifery, maternal and childcare [22]. The PHCS provided basic health services to one-fifth of the world’s population [23], and improved communicable disease control, including malaria, by using ‘mass mobilization’, public education, and auxiliary personnel training. The RCMS that was operated by the villagers on a collective and mutual aid basis effectively maintained activities of malaria prevention and treatment. The villagers received free medical care in the cooperative health stations [24].

Malaria epidemics occurred in many other parts of Yunnan Province where the activities of RCMS were sharply curtailed during the great culture revolution (Fig. 3). From 1968 to 1977, a population of 1,877,539 was affected by malaria epidemic in Yunnan alone and 1142 people died of malaria-related diseases; 409,117 malaria cases, 217.9 per 1000 person-years AMI and 26.11% (40,383/154,700) parasite prevalence was reported in the epidemic [11]. However, the RCMS provided malaria treatment and prevention, ensuring low and stable malaria prevalence in Hekou even though the county’s anti-epidemic station was combined into the county hospital and unable to fully collect and report on malaria cases.

Strong public health system is basis of for malaria control and elimination. China’s post-1978 economic reforms in agricultural production and public financing diminished collective financing and public support for the RCMS. The continuing decline in the RCMS seriously affected healthcare: the number of barefoot doctors per capita diminished; most barefoot doctors forewent continuing education; there was an increase in the financial burden borne by farmers; and hospitals experienced financial problems. With the collapse of RCMS financing, many barefoot doctors created private practices, charging patients for service and selling drugs [25, 26]. Accumulative impact of economic reforms contributed to the malaria epidemic in 1987. The epidemic and increasing malaria incidence attracted the attention of local government to invest for drugs and insecticides. However, financing and personnel problems in healthcare were not solved until after the epidemic of SARS in 2003. The epidemic of SARS urged a strong commitment from central government to public health, including financing and coordinating a national malaria control and elimination programme. Meanwhile, international investment (2003–2012) from the GFATM accelerated malaria control and elimination in China (Table 1).

High access of population to core interventions is crucial for effective control and elimination of malaria. During 1950–1960s when resources were short, the malaria burden reduced dramatically and put Hekou into pre-elimination stage. This is resulted from the high access of population to treatment and vector measures. From 2003, continuously high intervention moved it from pre-elimination into elimination (Table 1; Fig. 3). In spite of facing challenges today, such as resistance of anti-malarial drugs and insecticides [2], and the difficulty of finding a radical cure of vivax malaria [27], enough intervention could be effective. When five of the six GMS countries (all except China) reported artemisinin resistance of *P. falciparum* [28], the high coverage of intervention dramatically reduced the malaria burden [9], while drug sensitivity of malaria parasites has not changed significantly along the China-Myanmar border [29, 30].

Collaboration in prevention and control of cross-border malaria with neighbouring countries, and re-inforcement of early diagnosis and prompt treatment also contribute border malaria control and elimination. One of the major obstacles to achieving malaria elimination is movement across international borders. Strengthening surveillance activities for rapid identification of importation or re-introduction of malaria is essential to address the movement of malaria [10]. During American and Vietnam conflict from 1955 to 1975, China gave a full support to Vietnam for malaria control. The collaboration might contribute a stable malaria situation in Hekou County in the time. In 2010, collaboration and information exchange mechanism, including annual meeting, routine information exchange, migrant management was again established between China Hekou County and Vietnam Lao Cai Province for border malaria surveillance and control (Table 1) [31]. That overall incidence of malaria is low in Vietnam, where the most affected are the central and south central hilly and mountainous areas, and malaria transmission is interrupted in northern provinces [32, 33], would also benefit malaria elimination of Hekou County on the China–Vietnam border.

Malaria is associated with socio-economic development and environmental Change as well as effective interventions as documented in Hainan Province of China [34]. The close relationship between AMI and NGDP and NUR documented that facilitated malaria elimination in Hekou (Table 2; Fig. 4). Growth of GDP provided assurance of financial investment for malaria control and urbanization. Increasing production has been changing the biophysical environment. Increasing banana-growing areas reduce mosquito-breeding sites, and the use of insecticides kills mosquitoes directly. This is showed by the strong correlation between the AMI and BPA, especially during 2000–2013 when the BPA were increasing rapidly (Table 2; Fig. 4). Results of bilinear regression analysis indicate that socio-economic development and biophysical environment changes are associated with reduction of burden and elimination of malaria, as well as interventions, in Hekou County (Table 2; Fig. 4).

Four limitations exist in this study. Firstly, data of individual interventions and associated reduction of the malaria burden are not available, population level figures and qualitative descriptions are used as replacements. The results on malaria burden before 1951 was descriptive and were based on fragmentary descriptions. Similar limitation is that an ecological study existed in the study that was not able to prove impact given the lack of a control [35], only suggestive evidences can be provided. Secondly, from 1966 to 1986, malaria cases might have been under-reported, so that the true malaria situation cannot be documented (Table 1; Fig. 3). Thirdly, although the single and bilinear regression analysis documents that socio-economic development and environmental change contributes to reduced malaria burden and elimination, it cannot be used to document quantitative contributions from control interventions because of lack of sequential data of interventions. Fourthly, annually sequential data of GDP and urbanization for Hekou County are not available, so the national and provincial data are used for the linear regression analysis. However, Hekou is an underdeveloped border county in China so that investments from upper government, such as national and provincial governments, are the main resource for regional socio-economic development as well as malaria control. From this view, the national and provincial data for the analysis would work equally as the data from the county level.

## Conclusion

From the experiences of Hekou, five implications might be useful for global malaria eradication. First, political commitment and governmental executive ability are pivotal for malaria control and elimination. Under strong governmental commitment many socio-economic constraints (such as financial and human resources) could be overcome and healthcare systems can be strengthened. Second, national health orientations and policies can dramatically affect efficacy and interventions of malaria control. Third, integration of primary healthcare together with malaria control contributes to sustainability of effective malaria control and surveillance. Fourth, high coverage of malaria intervention and population access to the intervention is crucial at any stage of malaria control and elimination. Fifth, malaria incidence is affected by socio-economic development, biophysical environment change, and malaria situation of neighbouring countries or regions. Multifaceted factors, including political commitment, effective interventions, socio-economic development, and changing biophysical environment work together for the elimination of malaria in Hekou (Additional file 1).

## Additional file

**Additional file 1.** National gross domestic product (GDP) and urbanization rate (UR), Yunnan GDP and UR, Hekou’s banana planting area and annual malaria incidence.
