# Supplementary material for: Malaria from hyperendemicity to elimination in Hekou County on China–Vietnam border: an ecological study
Source: Malar J. 2017 Feb 7;16:66. doi: 10.1186/s12936-017-1709-z (PMC5297092; doi:10.1186/s12936-017-1709-z)
Supplement: Supplementary file 1 — Additional file 1. National gross domestic product (GDP) and urbanization rate (UR), Yunnan GDP and UR, Hekou’s banana planting area and annual malaria incidence. [file 12936_2017_1709_MOESM1_ESM.doc]

**The common caption for additional file: Data of malaria, socioeconomic development and banana planting**

Table1 National gross domestic product (GDP) and urbanization rate (UR), Yunnan GDP and UR, Hekou’s banana planting area and annual malaria incidence.

| Year | Population | No. malaria cases | Annual malaria incidence / 1000 person-years | National GDP (Billion CNY) | National UR (%) | Yunnan GDP (Billion CNY) | Yunnan UR (%) | Banana planting area (Hectares) |
| --- | --- | --- | --- | --- | --- | --- | --- | --- |
| 1952 | 18224 | 1076 | 59.04 | 67.9 | 12.5 | 1.3 | 7.8 | Na |
| 1953 | 18379 | 6591 | 358.62 | 82.4 | 13.26 | 1.6 | 8.22 | Na |
| 1954 | 20030 | 5208 | 260.01 | 85.9 | 13.37 | 1.6 | 8.29 | Na |
| 1955 | 20473 | 3382 | 165.19 | 91.1 | 13.48 | 1.7 | 8.36 | Na |
| 1956 | 22609 | 2373 | 104.96 | 102.9 | 14.44 | 2.0 | 8.95 | Na |
| 1957 | 23201 | 1142 | 49.22 | 106.9 | 15.4 | 2.0 | 10.8 | Na |
| 1958 | 28693 | 1005 | 35.03 | 130.8 | 16.85 | 2.5 | 12.7 | 0.13 |
| 1959 | 31977 | 1491 | 46.63 | 144 | 18.3 | 2.7 | 13.9 | 1 |
| 1960 | 33727 | 192 | 5.69 | 145.7 | 19.75 | 2.8 | 15 | 8 |
| 1961 | 31773 | 847 | 26.66 | 122.1 | 19.29 | 2.3 | 13.89 | 38 |
| 1962 | 34644 | 710 | 20.49 | 115.1 | 17.33 | 2.2 | 12.48 | 78 |
| 1963 | 35891 | 288 | 8.02 | 123.6 | 16.84 | 2.3 | 12.12 | 125 |
| 1964 | 37268 | 658 | 17.66 | 145.6 | 18.37 | 2.8 | 13.23 | 167 |
| 1965 | 39745 | 155 | 3.90 | 171.7 | 17.98 | 3.3 | 12.95 | 211 |
| 1966 | 43228 | 108 | 2.50 | 187.3 | 17.86 | 3.6 | 12.86 | 231 |
| 1967 | 45537 | 83 | 1.82 | 178 | 17.74 | 3.4 | 12.77 | 264 |
| 1968 | 47083 | 21 | 0.45 | 173 | 17.62 | 3.3 | 12.69 | 273 |
| 1969 | 50488 | 0 | 0.00 | 194.6 | 17.5 | 3.7 | 12.6 | 293 |
| 1970 | 53193 | 8 | 0.15 | 226.1 | 17.38 | 4.3 | 10 | 333 |
| 1971 | 58333 | 316 | 5.42 | 243.5 | 17.26 | 4.6 | 9.7 | 347 |
| 1972 | 58977 | 23 | 0.39 | 253 | 17.13 | 4.8 | 9.6 | 367 |
| 1973 | 61000 | 330 | 5.41 | 273.3 | 17.2 | 5.2 | 9.5 | 387 |
| 1974 | 63993 | 285 | 4.45 | 280.4 | 17.16 | 5.3 | 9.5 | 400 |
| 1975 | 67935 | 358 | 5.27 | 301.3 | 17.34 | 5.7 | 10.75 | 413 |
| 1976 | 63057 | 291 | 4.61 | 296.1 | 17.44 | 5.6 | 10.81 | 433 |
| 1977 | 64877 | 493 | 7.60 | 322.1 | 17.55 | 6.1 | 10.88 | 453 |
| 1978 | 69882 | 554 | 7.93 | 364.5 | 17.92 | 6.9 | 11.11 | 473 |
| 1979 | 75747 | 631 | 8.33 | 406.3 | 18.96 | 7.7 | 11.76 | 540 |
| 1980 | 73893 | 414 | 5.60 | 454.6 | 19.37 | 8.4 | 13 | 620 |
| 1981 | 66951 | 347 | 5.18 | 489.2 | 20.16 | 9.4 | 13.1 | 720 |
| 1982 | 67627 | 216 | 3.19 | 532.3 | 21.13 | 11 | 13.73 | 807 |
| 1983 | 68303 | 95 | 1.39 | 596.3 | 21.62 | 12 | 14.05 | 900 |
| 1984 | 68423 | 257 | 3.76 | 720.8 | 23.01 | 14 | 14.96 | 997 |
| 1985 | 67509 | 316 | 4.68 | 901.6 | 23.71 | 16.5 | 15.41 | 1077 |
| 1986 | 67457 | 370 | 5.48 | 1027.5 | 24.52 | 18.2 | 15.94 | 1150 |
| 1987 | 68279 | 2331 | 34.14 | 1205.9 | 25.32 | 22.9 | 16.46 | 1223 |
| 1988 | 68854 | 1018 | 14.78 | 1504.3 | 25.81 | 30.1 | 16.78 | 1317 |
| 1989 | 69478 | 722 | 10.39 | 1699.2 | 26.21 | 36.3 | 17.04 | 1383 |
| 1990 | 70488 | 287 | 4.07 | 1866.8 | 26.41 | 45.2 | 17.5 | 1457 |
| 1991 | 72260 | 319 | 4.41 | 2178.2 | 26.94 | 51.7 | 18 | 1483 |
| 1992 | 73653 | 299 | 4.06 | 2692.3 | 27.46 | 61.9 | 18.2 | 1523 |
| 1993 | 74309 | 424 | 5.71 | 3533.4 | 27.99 | 78.3 | 18.5 | 1533 |
| 1994 | 74767 | 458 | 6.13 | 4819.8 | 28.51 | 98.4 | 19 | 1583 |
| 1995 | 75767 | 258 | 3.41 | 6079.4 | 29.04 | 122.2 | 20 | 1600 |
| 1996 | 75958 | 227 | 2.99 | 7117.7 | 30.48 | 151.8 | 20.2 | 2180 |
| 1997 | 76473 | 150 | 1.96 | 7897.3 | 31.91 | 167.6 | 20.3 | 2753 |
| 1998 | 76965 | 104 | 1.35 | 8440.2 | 33.35 | 183.1 | 21 | 3320 |
| 1999 | 77509 | 179 | 2.31 | 8967.7 | 34.78 | 190 | 22 | 3900 |
| 2000 | 76837 | 178 | 2.32 | 9921.5 | 36.22 | 201.1 | 23 | 4500 |
| 2001 | 76837 | 145 | 1.89 | 10965.5 | 37.66 | 213.8 | 24.5 | 5073 |
| 2002 | 76256 | 75 | 0.98 | 12033.3 | 39.09 | 231.3 | 25.5 | 5640 |
| 2003 | 77015 | 89 | 1.16 | 13582.3 | 40.53 | 255.6 | 26 | 6213 |
| 2004 | 78154 | 149 | 1.91 | 15987.8 | 41.76 | 308.2 | 27 | 6800 |
| 2005 | 79185 | 101 | 1.28 | 18493.7 | 42.99 | 346.3 | 29.5 | 7373 |
| 2006 | 79444 | 126 | 1.59 | 21631.4 | 44.34 | 398.8 | 30.5 | 7933 |
| 2007 | 85753 | 58 | 0.68 | 26581 | 45.89 | 477.3 | 32 | 8527 |
| 2008 | 86615 | 16 | 0.18 | 31404.5 | 46.99 | 569.2 | 33.5 | 9133 |
| 2009 | 87260 | 16 | 0.18 | 34090.3 | 48.34 | 617 | 34.5 | 9733 |
| 2010 | 88307 | 18 | 0.20 | 39798.3 | 49.95 | 722.4 | 35.2 | 10000 |
| 2011 | 89451 | 10 | 0.11 | 48412.3 | 51.27 | 889.3 | 36 | 11200 |
| 2012 | 90073 | 1 | 0.01 | 53412.3 | 52.57 | 1030.9 | 39.31 | 12067 |
| 2013 | 90695 | 1 | 0.01 | 58801.8 | 53.7 | 1183.2 | 40.48 | 12200 |
| 2014 | 91319 | 0 | 0.00 | 63646.2 | 54.77 | 1281.5 | 41.73 | 12133 |
| 2015 | 92020 | 0 | 0.00 | 67670.8 | 56.1 | 1371.8 | 42 | 12067 |
